# Tuning the affinity of amphiphilic guest molecules in a supramolecular polymer transient network

**DOI:** 10.1039/d2ra00346e

**Published:** 2022-05-10

**Authors:** Maaike J. G. Schotman, Peter-Paul Fransen, Jiankang Song, Patricia Y. W. Dankers

**Affiliations:** Institute for Complex Molecular Systems, Eindhoven University of Technology P. O. Box 513 Eindhoven 5600 MB The Netherlands p.y.w.dankers@tue.nl; Department of Biomedical Engineering, Laboratory of Chemical Biology, Eindhoven University of Technology P. O. Box 513 Eindhoven 5600 MB The Netherlands; Department of Biomedical Engineering, Laboratory for Cell and Tissue Engineering, Eindhoven University of Technology P. W. Box 513 5600 MB The Netherlands

## Abstract

Dynamicity plays a central role in biological systems such as in the cellular microenvironment. Here, the affinity and dynamics of different guest molecules in a transient supramolecular polymer hydrogel system, *i.e.* the host network, are investigated. The hydrogel system consists of bifunctional ureido-pyrimidinone (UPy) poly(ethylene glycol) polymers. A monofunctional complementary UPy guest is introduced, designed to interact with the host network based on UPy–UPy interactions. Furthermore, two other guest molecules are synthesized, being cholesterol and dodecyl (c12) guests; both designed to interact with the host network *via* hydrophobic interactions. At the nanoscale in solution, differences in morphology of the guest molecules were observed. The UPy–guest molecule formed fibers, and the cholesterol and c12 guests formed aggregates. Furthermore, cellular internalization of fluorescent guest molecules was studied. No cellular uptake of the UPy–cy5 guest was observed, whereas the cholesterol–cy5 guest showed membrane binding and cellular uptake. Also the c12–cy5 guest showed cellular uptake. Formulation of the guest molecules into the UPy hydrogel system was done to study the guest–host affinity. No changes in mechanical properties as measured with rheology were found upon guest–hydrogel formulation. Fluorescence recovery after photobleaching showed the diffusive properties of the cy5-functionalized guests throughout the host network. The c12 guest displayed a relatively fast mobility, the UPy guest displayed a decrease in mobility, and the cholesterol–guest remained relatively stable in the host network with little mobility. This demonstrates the tunable dynamic differences of affinity-based interaction between guest molecules and the host network. Interestingly, the cholesterol guest is internalized in cells and is robustly incorporated in the hydrogel network, while the UPy guest is not taken up by cells but shows an affinity to the hydrogel network. These results show the importance of guest–hydrogel affinity for future drug release. However, if modified with cholesterol these guests, or future drugs, will be taken up by cells; if modified with a UPy unit this does not occur. In this way both the drug–hydrogel interaction and the cell internalization behavior can be tuned. Regulating the host–guest dynamics in transient hydrogels opens the door to various drug delivery purposes and tissue engineering.

## Abstract 2

Dynamicity plays a central role in biological systems, which can be mimicked by tuning dynamicity in hydrogel networks.

## Graphical abstract

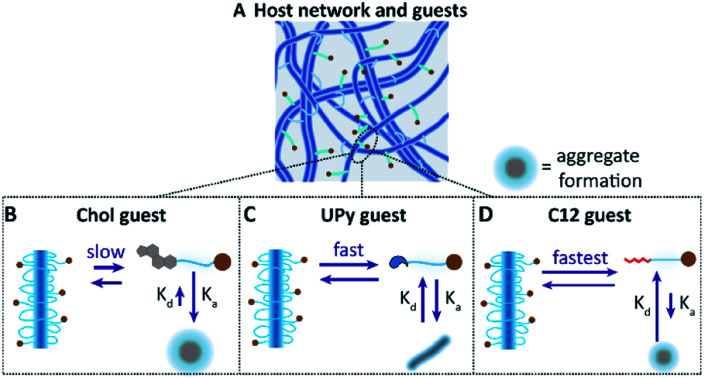

## Introduction

Biomaterials have increased in complexity and functionality over the last few decades, with many biomaterials being adaptable in degradability,^1^ bioactivity^2^ and drug release.^3^ Molecules such as polypeptides and polynucleotides have a diversity of structures and dynamics, displaying many unique properties.^4^ Based on reversible noncovalent interactions, *e.g.* hydrogen bonding, π–π interactions, electrostatic interactions, or hydrophobic interactions, natural systems can execute certain functions by altering their shape in place and time. The field of supramolecular chemistry, based on reversible noncovalent interactions between molecules, takes inspiration from these natural processes attempting to mimic its highly dynamic character. An example of such a supramolecular system is the peptide amphiphile, that can be crosslinked by hydrophobic and hydrogen bonding interactions,^5^ and can form a hydrogel in which the rigidity can be altered by, for example, changing the molecular sequence.^6^ Other types of amphiphiles, based on cholesterol or alkyl spacers, can induce membrane fusion of liposomes, which can play an important role in applications such as the fusion of liposomes and cells.^7,8^ Other examples of such systems that display reversible and dynamic properties are based on host–guest assembly,^9^ such as cyclodextrins,^10^ and curcurbiturils.^11^ A supramolecular hydrogel based on this cyclodextrin moiety was reported by Ooi *et al.*, who developed an alginate-based hydrogel functionalized with cyclodextrins (guests), to which different poly(ethylene glycol) (PEG) units functionalized with adamantane groups of different valencies were added.^12^ An increase in valency led to a change in dynamics, with an increase in binding affinity, and increase in storage modulus.

In our group we focus on the development of hydrogels based on specific stimuli-responsive supramolecular interactions, *i.e.* ureido-pyrimidinone (UPy) based hydrogels. These UPy moieties dimerize based on four-fold hydrogen bonding. The dimerization constant of the UPy-moiety in chloroform saturated with water was shown to be 1 × 10^7^ M^−1^.^13^ Conjugation of a urea units, flanked by hydrophobic alkyl spacers, can stabilize lateral stacking by hydrophobic interaction and formation of hydrogen bonding. Furthermore, introduction of a hydrophilic poly(ethylene glycol) (PEG) chain results in a bifunctional UPy–PEG hydrogelator (BF UPy–PEG).^14^ At higher concentrations in solution, *i.e.* c* the gelation concentration, a transient polymer network can be formed by fiber entanglement and physical crosslinks. The BF UPy–PEG hydrogel displays highly dynamic properties, with swift self-healing recovery (*K*_dim_ 1 × 10^7^ M^−1^ in chloroform saturated with water^13^). The exchange dynamics of the supramolecular interactions at different length scales can be tuned by differing the length of the alkyl spacer and PEG size.^14^ The adaptivity of these transient networks resemble the dynamic nature of biological structures such as the extracellular matrix, adapting to its environment over time. Previous work from our group displayed the exchange dynamics in solution to be controlled by mixing different guests, being mono- and bifunctional UPy units to host fibers.^15^ The dynamics of a UPy-based system in gel state was explored by encapsulating different monomeric and dimeric UPy–guest molecules in a UPy host hydrogel, displaying a robust interaction between the monomeric and dimeric UPy–guest molecules in the UPy–host network.^16^ Modular bioactivity could be implemented in a UPy-based hydrogel in a modular fashion, enabling cellular adhesion and growth on such transient networks.^17^ We described the conjugation strategy of a cholesterol moiety to a chemotherapeutic agent, which enhanced the affinity between the modified drug (guest) and the UPy-based hydrogel (host), resulting in a sustained guest release over time.^18^ In another study, the dynamics of a cholesterol-conjugated siRNA moiety within the UPy-based hydrogel were explored, displaying a relatively slow diffusion in comparison to the siRNA containing no cholesterol-conjugation.^19^ This further elucidates the enhanced affinity between the guest (cholesterol modified siRNA) and the host (UPy-based hydrogel).

**Fig. 1:**
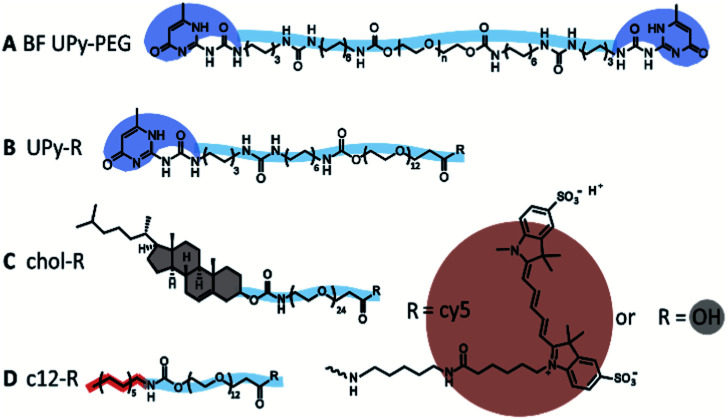
Schematic overview of the molecules used in this study. The chemical structure of the hydrogelator BF UPy–PEG (A), the monofunctional UPy guest (B), the cholesterol guest (C), and the dodecyl c12 guest (D), modified with cy5 or unmodified (–OH).

In this study, we further explore the dynamics between different guests, and a supramolecular BF UPy–PEG host hydrogel. We examined three different guest molecules, hypothesized to have distinctive affinities with the UPy-based host hydrogel (Fig. 1A). The monofunctional UPy unit functionalized to an oligo(ethylene glycol) (OEG) was presented as an guest molecule (UPy–COOH, Fig. 1B), interacting with the BF UPy–PEG hydrogel by complementary four-fold hydrogel bonding. A dodecyl molecule and cholesterol molecule were functionalized to an OEG (c12–COOH and chol–COOH, respectively, Fig. 1C and D), resulting in two amphiphilic compounds. These two guest molecules are hypothesized to interact with the host BF UPy–PEG based on hydrophobic interactions between the guests and the hydrophobic pockets present in the hydrogel network, created by the lateral stacking of the alkyl spacers induced by the urea moieties (hydrogen bonding) and hydrophobic interaction of the alkyl spacers in the BF UPy–PEG backbone. The unmodified guest molecules were examined (with a carboxylic acid end-group; a stable, versatile chemical handle for functionalization) on assembly behavior in solution, and effect on rheological properties upon addition to the hydrogel. The guest assembly in solution was studied with cryogenic transmission electron microscopy (cryo-TEM), and functionalization of a fluorophore (cy5) enabled visualization of cellular uptake and diffusivity throughout the hydrogel network using fluorescence recovery after photobleaching (FRAP).

## Results and discussion

### Guest molecules in solution

**Fig. 2:**
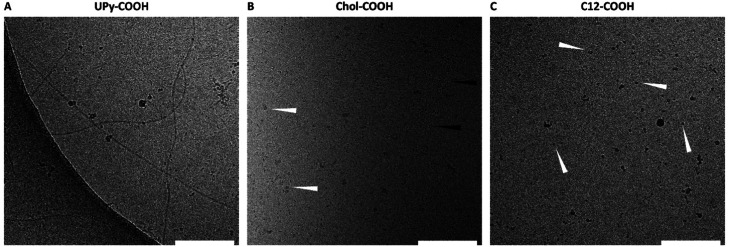
Transmission electron micrographs of the UPy–COOH guest (A), the chol–COOH guest (white arrows indicate relatively large and black arrows indicate small aggregates) (B), and c12–COOH guest (white arrows indicate small aggregates) (C). All guest molecules were measured at a concentration of 50 μM in PBS/DMSO (95/5 v/v%), at a magnification of 240 00× (scale bar represents 200 nm).

To elucidate the assembling behavior in solution, cryo-TEM measurements were performed. Here, the unfunctionalized guest molecules with the carboxylic acid side-group were measured at a concentration of 50 μM. The UPy–COOH displayed single fibers morphology as well as patches of fibers (Fig. 2A), indicating the clear fibrous self-assembling pattern of UPy-modified molecules. For chol–COOH, small aggregates and micellular formation can be observed (Fig. 2B). Occasionally, chol–COOH showed to form small fibrils, which are hypothesized to be wormlike micelles. However, larger dispersed aggregates were formed as well, too high in size to be observed in cryo-TEM (μ-sized particles). Dynamic light scattering showed high polydispersity of the sample in the microscale range (data not shown), further confirming that cryo-TEM does not represent the full structural overview of the particle in solution. For c12–COOH, small spherical aggregates, likely to be micellular formation, were observed (Fig. 2C).

A cy5 fluorophore was coupled to the guest–molecules to allow for visualization *via* fluorescence. Briefly, the cy5-labelling of the three different guests was carried out by reacting the carboxylic acid of the guests to the primary amine present on the cy5 molecule. HATU was used as a coupling reagent in combination with a base to activate the carboxylic acid, subsequently followed by cy5 addition for the carboxamide formation. After purification of the cy5-functionalized guests, the compounds were dissolved in DMSO, and mixed in the desired ratio to the medium (cellular uptake studies) or hydrogelator (FRAP experiments) for proper dissolution. The DMSO content of the final solution was kept below 5%.

**Fig. 3:**
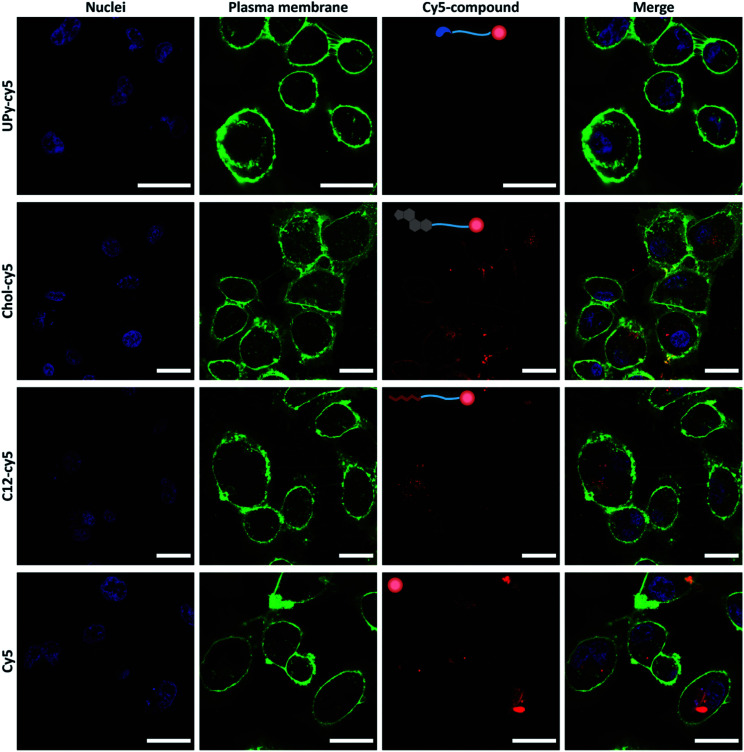
Confocal micrographs of HK-2 live cells showing the cy5-labelled guest uptake studies. With the nuclei (blue), plasma membrane (green), cy5 compounds (red), and the merged image visualized. The guests were added to medium at a concentration of 10 μM for a time span of 2 hours, whereafter the cells were washed and visualized by live-cell imaging (the scale bar represents 30 μm).

The cellular uptake of the cy5-functionalized guests was examined on an immortalized proximal tubule epithelial cell line from normal adult human kidney (HK-2). Each guest was added to cell medium at a concentration of 10 μM and incubated for 2 hours with the cells, whereafter the cells were washed, stained and imaged by confocal microscopy under the same settings (Fig. 3). Differences in cellular uptake of the guest molecules was observed, with the UPy–cy5 compound showing a minimum to no cellular uptake. From previous work, presence of cationic charges increased the cellular uptake, whereas neutral charge showed no binding or permeation.^20^ The monofunctional UPy–cy5 guest, having no cationic charge present, display cellular inertness. The chol–cy5 is observed to bind to, as well as permeate the membrane, indicating a significant effect of the chol–cy5 guest on the cellular interaction. The chol–cy5 is clearly visible on the membrane, with cholesterol playing a role in the regulation of membrane fluidity, permeability, and hydrophobicity.^21^ Furthermore, cholesterol plays an important role as signal transducer and solubilizer of other lipids within the cell.^22^ This makes cholesterol-modification interesting in the field of drug delivery, in which cholesterol moieties are often used to enhance the cellular uptake.^18,23,24^ c12–cy5 shows intracellular uptake, with clusters c12–cy5 appearing to be present in the cytosol of the cell. Functionalization with alkyl spacers has showed to enhance the cellular transfection, due to its interaction with the cellular membrane.^25–27^ The cellular uptake is hypothesized to take place by transmembrane lipid translocation (flip–flop), with diffusion and interaction with the cellular membrane playing a significant role.^28^ However, solely cy5 also shows cellular uptake, with large intracellular clusters being visible intracellular, as shown in previous studies.^29^ This indicates that the cy5 can have an effect on the cellular uptake of the cy5–bound guests. Still, clear differences are observed within the cy5-labelled guest molecules, with UPy–cy5 showing no uptake, and c12–cy5 and chol–cy5 displaying cellular uptake, with the latter one also displaying membrane binding. In previous work, we showed that using cholesterol or UPy-moieties, the retention of the guest molecules in the gel can be steered, whereafter these moieties can be taken up by the cells (cholesterol), or not taken up by the cell (UPy) after release.^18,30^ The properties of the guest molecules were further explored by implementation in a UPy-based hydrogel.

### Guest molecules in the hydrogel system

**Fig. 4:**
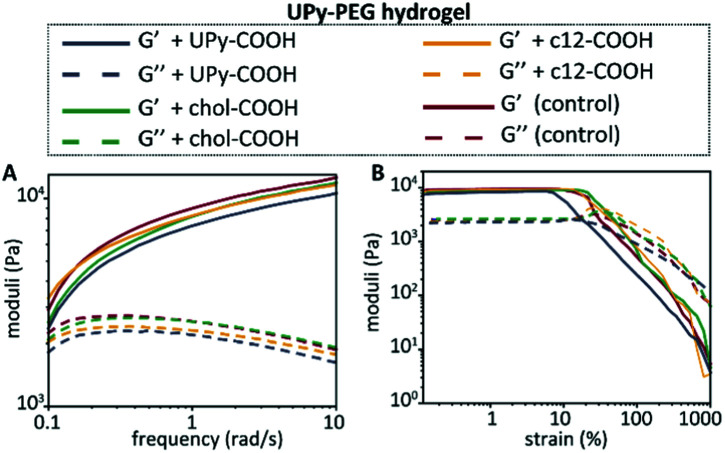
Mechanical hydrogel properties of the BF UPy–PEG hydrogel upon anchor addition, with the frequency sweep at 1% strain (A), and the strain sweep at 1 rad s^−1^ (B) measured at 37 °C.

Hydrogels of the BF UPy–PEG were formed by dissolving the host polymer at 10 wt% concentration at basic conditions (pH 11.7), which resulted in a viscous liquid. The guest molecules were added from a DMSO stock solution while in the viscous liquid state, and by neutralization a hydrogel was obtained. The mechanical properties of the host hydrogel were examined upon guest encapsulation by rheology (Fig. 4). The final concentration of the guest molecules in the hydrogel was 100 μM, and a control was measured containing an equal amount of DMSO (5 v/v%) added to the host hydrogel. Frequency and strain sweeps were measured, that displayed an increase in storage moduli with an increase in frequency for all hydrogels. This indicates an increase in elastic properties at higher frequencies, whereas at lower frequencies the viscous properties of the hydrogel are increasing, with the viscous properties dominating <0.1 rad s^−1^. Upon anchor addition, similar frequency responses were observed, with addition of chol–COOH, c12–COOH, and UPy–COOH displaying a storage modulus of 2540 Pa, 3310 Pa, and 2370 Pa at 0.1 rad s^−1^, respectively. At 10 rad s^−1^, the modulus increases to 11 900 Pa, 11 700 Pa, and 10 600 Pa, respectively. A slow decrease was observed for the loss modulus when the frequency was increased, indicating a less predominant viscous behavior at higher frequencies.

The strain-sweep displayed similar storage and loss moduli for all hydrogels, with the storage moduli showing small variations between each condition in the linear regime (∼9500 Pa for the control, ∼8400 Pa for UPy–COOH, ∼9000 Pa for chol–COOH, and ∼9400 for c12–COOH). The determined yield stresses based on the strain sweeps are in similar regimes, with 15.5 Pa, 14.4 Pa, 23.7 Pa, and 9.0 Pa for the control, chol–COOH, c12–COOH, and UPy–COOH, respectively. These results confirm the viscoelastic properties of the hydrogels, with the guest molecule addition not showing significant changes in mechanical properties of the host hydrogel.

The calculated partition coefficient (*c* log *P*) values display an indication on the lipophilicity of the compounds. The *c* log *P* values for the guests were 2.2 for UPy–COOH, 6.0 for chol–COOH, and 3.6 for c12–COOH. The hydrophilicity of the UPy–COOH guest to be the highest, whereas the chol–COOH guest showed to exhibit a more hydrophobic character. While the chol–COOH guest was well-dissolved in the hydrogel host at a concentration of 100 μM (5% DMSO), similar amounts of chol–COOH in PBS show precipitation and therefore poor solubility. We hypothesize that this is due to the increased affinity with the BF UPy–PEG network, with the hydrophobic pockets present in the host network increasing the solubility of the chol–COOH.

**Fig. 5:**
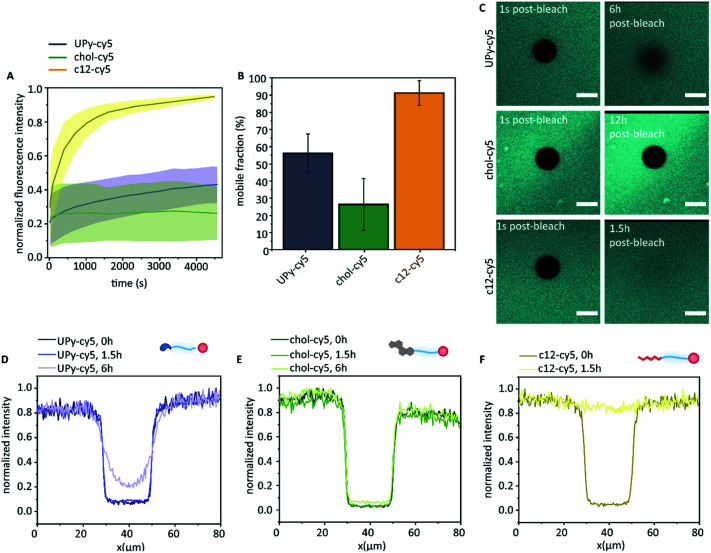
Exchange dynamics of the guests in the host hydrogel. The normalized fluorescence intensity of the different cy5 functionalized guests after photobleaching (A), and the fraction of fluorescence intensity that recovers when fluorescence intensity reaches a plateau (B, mobile fraction), data is represented as ±SD, *n* = 3. The confocal micrographs of the different cy5-labeled guest molecules directly post-bleaching, and the UPy–cy5 visualized 6 h post-bleaching, the chol–cy5 visualized 12 h post-bleaching, and c12–cy5 visualized 1.5 h post-bleaching (C, scale bar represents 20 μm). The measured intensity profile for the bleached spots after 0 hour, 1.5 hour, and 6 hours post-bleaching of (D) UPy–cy5, (E) chol–cy5, and (F) c12–cy5.

The molecular dynamics of the guest in the host network was examined by FRAP (Fig. 5). Here, the cy5-labelled guest molecules were bleached by high-intensity illumination at a selected region of 20 μm in diameter. This resulted in a clear dark circular spot, of which the diffusion of the cy5-labelled guest molecules towards the bleached spot was measured over time (Fig. 5C). Confocal microscopy displayed homogeneous fluorescence of the c12–cy5 guest, as well as the UPy–cy5 guest. The chol–cy5 guest showed an overall homogeneous distribution in the hydrogel, where intermittently small micron-sized aggregates were observed (up to ∼3 μm).

The FRAP data indicates a clear difference in diffusive behavior of each guest throughout the hydrogel, of which the swiftest recovery was observed for the c12–cy5 guest, reaching a plateau value after approximately 70 minutes with a mobile fraction of 0.91 ± 0.07. The half time recovery was determined by fitting the data with a single exponential growth model, obtaining a half-time recovery of 4.45 ± 3 min. This indicated that, while the c12–cy5 guest was hypothesized to remain in the hydrophobic pockets of the UPy–PEG hydrogel network, there was a mobility of this guest within the host network, confirmed by measured intensity profiles (Fig. 5F), that display a restored recovery after 1.5 hours. The cy5 guest was measured as a control, which displayed too swift recovery for measuring (sub-second time scale recovery). The diffusive mobility of UPy–cy5 was observed to be slower, with a plateau reached after approximately 4 hours, displaying a mobile fraction of 0.56 ± 0.11. After 6 hours, the bleached spot was observed to not be fully recovered (Fig. 5C). A 100% recovery is not obtained here, which is consistent with previously experiments.^16^ The half-time recovery was determined to be 53 ± 20 min. The lowest mobility was observed for chol–cy5, displaying a mobile fraction of 0.26 ± 0.15. This was confirmed by the intensity profile, showing a limited fluorescence recovery even after 6 hours. A single exponential fit could not be performed on this data, due to the low initial recovery leading to an improper fit. From this data, we can conclude that all guest molecules show an affinity with the host hydrogel network, all leading to a slow diffusion (in comparison to the cy5 control). The fastest diffusion in the host network is displayed by the c12–cy5 guest molecule, whereas the chol–cy5 displays the lowest mobility throughout the network, where a limited recovery is observed even after 12 hours (Fig. 5C). The UPy–Cy5 guest molecule displays a lower diffusion in the host network compared to the c12–cy5, indicating a higher affinity with the host network in comparison to the c12–cy5. These results support our hypothesis, that different dynamics are obtained when guest molecules are introduced to the host network, with different affinity based-interactions (hydrogen-bonding and hydrophobic host–guest assembly).

**Fig. 6:**
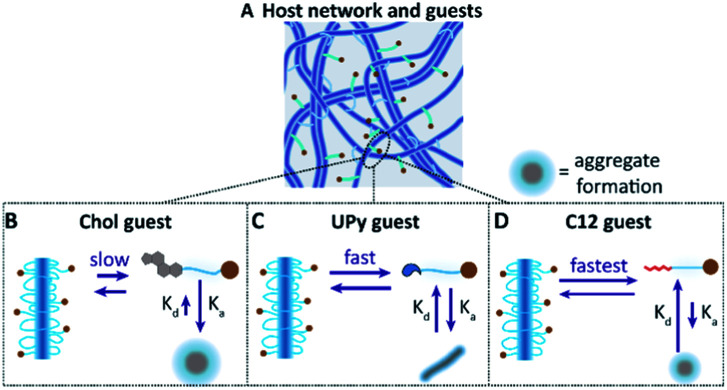
Hypothesized interaction mechanism between the host network and guest molecules (A), displaying the slow equilibrium of intercalation of the chol guest with the host network (B), whereas the association constant towards the aggregate formation within the network is hypothesized to be equal to the exchange of guest molecules within the host network (C). The fastest exchange is hypothesized to be between the c12 guest and host network, which displays higher mobility and exchange dynamics within the host network (D).

While an interaction between the chol–cy5 guest and the hydrophobic spacers of the host network is hypothesized, the possibility of aggregate formation within the host network remains likely for the high immobile state of the chol–cy5 guest (Fig. 6B). High polydisperse aggregate formation was observed in PBS by dynamic light scattering (results not shown), and small micron-sized aggregates were observed in the confocal micrographs of the chol–cy5 guest within the host hydrogel network (Fig. 5C), which can significantly limit the mobility of the guest molecules. An increased association constant for aggregate formation is hypothesized, with dissociated chol–cy5 guests displaying a slow exchange within the host hydrogel network. An increase in solubility of the cholesterol guest in the host hydrogel is shown in comparison to PBS. We hypothesize an interaction with small cholesterol aggregates within the hydrophobic domains present in the host network, improving the solubility within the hydrogel network.

A faster exchange was observed between the UPy guest and the host network, with intra and inter-fiber diffusion, as reported in a previous study.^16^ The UPy guest shows to form elongated fibers in PBS (Fig. 2A), as well as micelles.^31^ Therefore, the possibility of aggregate or fiber formation of solely the UPy guest molecules, thereby limiting the diffusion, remains a possibility (Fig. 6C). Therefore, whether diffusion of a single UPy guest molecule, or diffusion of the entire fiber containing the fixed guest molecules slows down fluorescent recovery remains unclear.

The fastest exchange of the three introduced guest molecules was displayed by the c12 guest, which is hypothesized to display fast exchange dynamics with the host network. Due to a small hydrophobic spacer (c12), the guest molecule is hypothesized to have the lowest binding affinity with the host network, remains in the host network only shortly and displaying a fast exchange (Fig. 6D). Presence of small c12 guest aggregates, as displayed in the cryo-TEM images (Fig. 2C), is possible, which can limit the guest mobility. The binding interaction of these guest molecules can be further explored by examining the release profile from the host hydrogel network, *e.g.* with LC-MS/MS. Overall, these results show the complexity of the possible interaction mechanisms and dynamic behavior of guest molecules within the host network.

## Conclusions

This study demonstrates tunability of guest–hydrogel affinity using hydrogen-bonding, and hydrophobic interactions. Three different supramolecular guests molecules were explored, which displayed differences in nanoscale assembly, cellular uptake and dynamicity within the host supramolecular polymer hydrogel. Future research into the dynamic adaptivity of implemented bioactive properties can adapt the cell-adhesive properties of supramolecular polymer hydrogels, whereas release kinetics of these functionalization modes can enhance drug efficacy. This establishes the basis of a generic ‘plug-and-play’ system to tune the bioactive properties of hydrogels, and release rate of a wide variety of drug molecules, based on the requirements for the biomedical application or disease in scope.

## Experimental section

### Materials and instrumentation

All reagents and chemical were obtained from commercial sources and used without purification unless stated otherwise. Phosphate buffered saline (PBS) tablets, 4-methylmorpholine (MMP), *N*,*N*-diisopropylethylamine (DIPEA) and 1-[bis(dimethylamino)methylene]-1*H*-1,2,3-triazolo[4,5-*b*]pyridinium 3-oxide hexafluorophosphate (HATU) were purchased from Sigma-Aldrich. Sulfo-Cyanine5 amine was purchased at Lumiprobe. H_2_N–PEG_24_–CO–OtBu was purchased from Iris Biotech. The UPy–PEG polymer with *M*_n,PEG_ = 10 kg mol^−1^ was synthesized by SyMO-Chem BV, Eindhoven. Nunc™ Lab-Tek™ Chambered Coverglass (8-well) were purchased at ThermoFisher Scientific. A Grace Reveleris X2 Flash Chromatography System using Reveleris Silica Flash Cartridges was used for automated column chromatography. Reverse-phase high-performance liquid chromatography-mass spectrometry (RP-HPLC-MS) was performed on a Thermo scientific LCQ fleet spectrometer. Rheological measurements were performed on an Anton Paar Physica MCR501 rheometer. ^1^H-NMR and ^19^F-NMR spectra were recorded on a 400 MHz NMR (Varian Mercury Vx or Varian 400 MR) operating at 400 MHz. FRAP measurements were performed on a Leica TCS SP5 inverted confocal laser scanning microscope. The clogP values were determined by Chemdraw. A Tecan Spark 10 M plate reader was used for analysis of the cy5 fluorescence.

### Synthetic procedures

#### Synthesis of c12–COOH

**Scheme 1:**
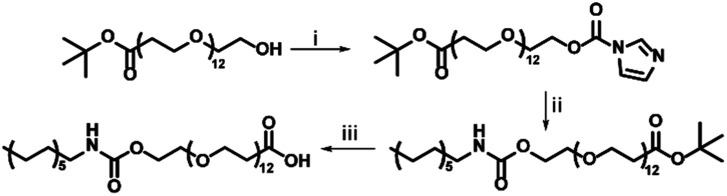
Synthesis of c12–COOH. (i) CDI, CHCl_3_, RT, overnight. (ii) C_12_H_27_N, CHCl_3_, reflux at 60 °C, overnight, (iii) TFA, DCM, room temperature, 2 h, 73%.

OEG_12_–*t*Bu (146.6, 0.232 mmol) was dissolved in 2 mL chloroform, which was added dropwise to a solution containing *N*,*N*-carbonyldiimidazole (207 mg, 1.27 mmol) in 2 mL chloroform under stirring. This was left stirring overnight at room temperature. After extraction with aqueous citric acid (2 mL), the organic phase was obtained. Analysis with RP-LC-MS revealed complete conversion. Dodecylamine (204.2 mg, 1.01 mmol) dissolved in CHCl_3_ was added dropwise to the reaction mixture under stirring conditions. The reaction mixture was stirred overnight at 60 °C overnight at reflux. Extraction by aqueous citric acid addition (2 mL) led to a milky solution. Subsequent extraction with brine was performed (3 × 4 mL), resulting in a clear organic layer. The product was further purified by column chromatography using silica, eluting with chloroform containing 5% ethylene glycol dimethacrylate, and a gradient of methanol from 0 to 10%, and a run time of 15 minutes. RP-LC-MS confirmed a pure product. Solvent were removed using a rotary evaporator, after which dichloromethane (DCM) was added to the product (5 mL), to which trifluoroacetic acid (TFA, 5 mL, 1 : 1 v/v%) was added. This was stirred for 2 hours at room temperature, whereafter DCM was removed by rotary evaporation. TFA was removed from the solution by coevaporation with toluene (3 × 8 mL toluene, 1 × 50 mL toluene) performed by rotary evaporation. This was confirmed with ^19^F-NMR, yielding a pure product (141.2 mg, 0.17 mol, 73%) (Scheme 1).

#### Synthesis of c12–cy5

c12–COOH (1.7 mg, 0.0021 mmol) was dissolved in DMF (1 mL), and HATU (1.57 mg, 0.004 mmol) was dissolved in DMF (1 mL) whereafter the HATU was added dropwise to the c12–COOH solution. MMP (1.8 μL, 0.017 mmol) was added to the solution, which was stirred for approximately 10 minutes. Sulfo–Cy5–NH_2_ (1.9 mg, 0.0027 mmol) was added to the solution dissolved in DMF (3 mL). This was stirred under argon conditions for 4 hours. LC-MS was used to confirm the formation of the reaction product. The crude product was purified by automated reversed-phase C18 silica (4 g), at a flow rate of 18 mL min^−1^. The eluents used were H_2_O : ACN (95 : 5 till 100% of ACN in 15 minutes). Freeze–drying of the collected fractions yielded pure c12–cy5 (1.8 mg, 55% yield). ESI-MS: *m*/*z* calc. for C_78_H_128_N_5_O_22_S_2_: 1550.85; obs. [M + 2H]^2+^ 775.33, [M + H]^+^ 1552.00.

#### Synthesis of chol–cy5

Chol–PEG_24_–COOH was synthesized as previously reported.^18^ Chol–PEG_24_–COOH (5 mg, 0.0032 mmol) was dissolved in DMF (1 mL) and HATU (2.5 mg, 0.0065 mmol) was added to the mixture. DIPEA (3.5 μL, 0.0201 mmol) was added to the mixture and stirred at room temperature for 15 minutes. Sulfo–Cy5–NH_2_ (3.1 mg, 0.0042 mmol) was added dropwise to the mixture and left stirring under argon condition at room temperature overnight. The following day, the reaction mixture was washed with brine two times, after which further purification by automated reversed-phase C18 silica (4 g) gel column chromatography was performed (flow rate 15 mL min^−1^, eluent: H_2_O : THF 95 : 5 until 100% of THF in with reverse chromatography was performed in 15 minutes). Due to further impurities, dialysis was performed using a MWCO of 500–1000 Da for 3 days against demi water. After dialysis, the sample was lyophilized, resulting in a pure blue powder (2.5 mg, 34% yield). HRMS (MALDI-TOF): *m*/*z* calculated for C_117_H_196_N_5_O_34_S_2_: 2280.32; found 2280.41 [M + H]^+^.

#### Synthesis of UPy–cy5

The UPy–OEG_12_–COOH precursor was synthesized as previously reported.^32^ UPy–OEG_12_–COOH (2.36 mg, 0.00208 μmol) was dissolved in DMF (1 mL) and HATU was added to the mixture (1.58 mg, 0.00416 mmol). *N*,*N*-Diisopropylethylamine (2.15 mg, 16.6 μmol) was added and the solution was stirred at room temperature for 15 min. Sulfo–Cy5–NH_2_ (2 mg, 27.0 μmol) dissolved in DMF (3 mL) was added to the solution and stirred for 1 h at an argon environment. H_2_O (containing 0.1 v/v% formic acid, 20 mL) was added to the solution and centrifugated (4 min, 3000 rpm) followed by decantation. Ultrapure water was added (20 mL) and the product was lyophilized. The compound was purified with preparative RP-HPLC using a gradient of 40% ACN in H_2_O (both containing 0.1 v/v% formic acid). Lyophilization yielded pure 3 (1.75 mg, 9.4 μmol, 45%) blue solid. ESI-MS: *m*/*z* calc. for C_91_H_149_N_11_O_25_S_2_ 1861.37; obs. [M + 3H]^3+^ 621.33, [M + 2H]^2+^ 931.17, [M + H]^+^ 1861.75.

### Cryo-TEM measurements

For cryo-TEM measurements quantifoil carbon covered grids were used (Electron Microscopy Sciences, 200 mesh, 50 μ hole size). Prior to sample addition, grids were surface plasma treated (at 5 mA for 40 s) using a Cressington 208 carbon coater. Using an automated vitrification robot (FEI Vitrobot™ Mark III), 3 μL sample was applied to the grids and excess sample was removed by blotting using filter paper for 3 s at −3 mm. The thin film formed was vitrified by plunging the grid into liquid ethane just above its freezing point. On an FEI-Titan TEM equipped with a field emission gun operating at 300 kV the samples were examined. Post-GIF (Gatan imaging filter) 2 × 2 Gatan CCD camera was used for recording of the images. Micrographs were taken at low dose conditions, using a defocus setting of −5 μm at 25k magnification, or defocus setting of −40 μm at 6.5k magnification.

### Cellular experiments

Human kidney 2 cells (HK-2; ATCC, Germany), immortalized by transduction with human papilloma virus 16 E6/E7 gene, were cultured at 37 °C in 95% air/5% CO_2_ atmosphere, in DMEM (ref. 22320-022; Gibco, UK) supplemented with 10% Fetal Bovine Serum (FBS; Greiner Bio-one, The Netherlands), and 1% penicillin/streptomycin (P/S; Invitrogen, USA). The cells were passed twice a week. For the aggregate uptake studies, HK-2 cells were seeded in an 8-well Thermo Fisher Scientific™ Nunc™ Lab-Tek™ Chamber with #1 borosilicate glass bottom at a density of 2.5 × 10^4^ cells cm^−2^ cells per well (*n* = 4). The guests (UPy–cy5, chol–cy5, c12–cy5) were prepared by adding the necessary amounts from stock solution (5 mg mL^−1^ in DMSO) to medium at a concentration of 10 μM. This was left at room temperature while being stirred overnight. The cells were washed three times with PBS after overnight attachment and 0.4 mL of the guests molecules in full culture medium suspension was added to each well. This was incubated for 2 hours, after which the cells were washed with PBS. Subsequentially, cellular nuclei were stained with Hoechst 33342 (Thermofisher Scientific) and cellular membrane was stained with CellMask™ Green plasma membrane stain (Thermofisher Scientific). After staining, the cells were washed three times with PBS, after which Invitrogen™ Live Cell Imaging Solution was added to each well for live imaging. The live imaging was performed under a Leica TCS SP5 inverted confocal laser scanning microscope at 37 °C.

### Hydrogel preparation

Hydrogels were prepared with a final concentration of 10 wt% polymer content, where the polymer was firstly dissolved in a basic PBS solution (pH 11.7, using 1 M NaOH) at a temperature of 70 °C, being stirred for 1 hour. Subsequently, the guests molecules were added from stock solution with a final concentration of 40 μM for FRAP measurements. For rheological measurements, guests molecules were added from a stock solutions, with a final concentration of 100 μM in the hydrogel. The solutions was mixed for 15 minutes at room temperature, whereafter the sol-state solutions were pipetted in an 8-well Thermo Fisher Scientific™ Nunc™ Lab-Tek™ Chamber for FRAP measurements. For rheological measurements, the hydrogelators (100 μL) were pipetted in a cylindrical Teflon mold (diameter 8 mm, 2 mm height). By pH-induced gelation, the hydrogels were prepared in the wells upon addition of 1 M HCl (1.4 μmol per 100 μL gel solution). This was equilibrated for 1–2 hours before measuring.

### Rheological measurements

Hydrogels were measured at 37 °C using a 8 mm plate–plate with a distance of 1 mm on an Anton Paar Physica MCR501 rheometer. Low viscosity silicon oil (47 V 100 m RHODORSIL®) was used to surround the hydrogels to prevent water evaporation. A time sweep was performed, with the storage and loss moduli were recorded for 10 minutes at 1% strain, 1 rad s^−1^, whereafter the angular frequency (100 to 0.1 rad s^−1^, 22 measurement points) at 1% strain and strain sweep (1 to 1000%, 22 measurement points) at 1 rad s^−1^ were recorded. Each condition is measured in duplicate to confirm reproducibility, whereafter one representative measurement is plotted. The yield stress was determined by measuring the strain-sweep of each hydrogel, from which the cross-over point between the linear regime and a power fit plot for the final 10 points of the curve (strain 145–1000%) was determined, obtaining the yield stress.

### Fluorescence recovery after photobleaching

A 20× objective (HCX PL APO CS 20.0 × 0.70 DRY UV) was used for imaging and the hydrogels were prepared in an 8-well Thermo Fisher Scientific™ Nunc™ Lab-Tek™ Chamber by pH-induced gelation. Surrounding empty wells were filled with MilliQ to prevent drying of the gels. The sample was placed inside an environmental chamber at 37 °C. The exchange dynamics were examined by illumination of the white laser at 646 nm excitation and 660–700 nm emission with a hybrid detector. The bleached circular area of the hydrogel was kept constant at 20 μm, and illumination at a laser power of 60% was performed for 10 frames (1.3 s per frame). Post-bleaching images were taken over a time-span of 2–12 hours, depending on the added anchor. The data was normalized by dividing the average gray values of the bleached area by the average gray values of the total area. Using the FRAPbot software,^33^ the mobile fraction was determined by single exponential fitting. FRAP measurements were performed in triple. Using the imageJ software, the normalized intensity profile of the circular diameter was determined for each anchor condition directly post-bleaching, 1.5 hours post-bleaching, and 6 hours post-bleaching.

## Author contributions

P. Y. W. D. and M. J. G. S. conceived and designed the experiments. P. P. K. H. F. and M. J. G. S. performed the synthetic procedures. J. S. and M. J. G. S. performed the cellular experiments. M. J. G. S. and P. Y. W. D. wrote the manuscript, which was edited by all co-authors.

## Conflicts of interest

There are no conflicts to declare.

## Supplementary Material
